# Supplementary material for: Leaving no one behind in health: Financial hardship to access health care in Ethiopia
Source: PLoS One. 2023 Mar 13;18(3):e0282561. doi: 10.1371/journal.pone.0282561 (PMC10010508; doi:10.1371/journal.pone.0282561)
Supplement: S1 Text — (DOCX) [file pone.0282561.s001.docx]

## S1 Text: Survey Questionnaire (English version)

Household Code____________ Date of interview______________

Interviewer name______________ signature________________

Checked by ______________ signature________________

**Part I: demographic and Socio economic characteristics of the household**

1. Sex of the household head? 1. Male 2. Female
2. Age of the household head? Years old
3. Religion of the household head? 1. Orthodox 2. Muslim 3. Protestant 4. Others specify……
4. What is your ethnicity? 1. Amhara 2. Oromo 3. Tigre 4. Others specify…………
5. Marital status of the household head? 1. Single 2. Married 3. Separated 4. Divorced 5. Widowed
6. Educational status of the household head? 1. Can’t write and read 2. Read and write only with no formal education 3. Primary (1-8) 4. Secondary (9-12) 5. College/university and above
7. Occupation of the household head? 1. Currently unemployed 2. Self-employed 3. Government employed 4. Working in private sector 5. Others specify……
8. What is your average monthly income? ETB
9. Number of household members (including the head)? , list (not head) them the table below.

| Code | R/ship to head | Age | Sex | Religion | Educational status | Occupation | Av. Income/month |
| --- | --- | --- | --- | --- | --- | --- | --- |
| M1 |  |  |  |  |  |  |  |
| M2 |  |  |  |  |  |  |  |
| M3 |  |  |  |  |  |  |  |
| M4 |  |  |  |  |  |  |  |
| M5 |  |  |  |  |  |  |  |
| M6 |  |  |  |  |  |  |  |
| M7 |  |  |  |  |  |  |  |
| M8 |  |  |  |  |  |  |  |
| M9 |  |  |  |  |  |  |  |
| M10 |  |  |  |  |  |  |  |
| Total |  |  |  |  |  |  |  |

**Part II: Household Wealth index measuring variables**

| s/n | | Questions | Answers | | s/no | Questions | Answers |
| --- | --- | --- | --- | --- | --- | --- | --- |
|  | | Ownership of the household | 1. Private 2. Rent | |  | House’s main floor material? | - - - 1. Natural(soil       2. Concrete       3. Ceramics       4. Others specify….. |
|  | | Main source of drinking water? | 1. Pipe water in dwelling 2. Protected dug well 3. Unprotected dug well 4. Protected spring 5. Others specify…… | |  |  |  |
|  | | Type of latrine? | 1. Not at all 2. Traditional pit latrine 3. VIP 4. Pour and flush 5. Others specify….. | |  | House’s main roof material? | - - - 1. Thatch roof       2. Corrugated iron sheet       3. Other (specify)______ |
|  | | Type of cooking fuel? | 1. Wood 2. Charcoal   3. Biogas 4. Kerosene  5.Electricity | |  | Household main wall material? | - - - 1. Wood with Mud       2. Concrete       3. Ceramic       4. Other (specify)______ |
| Does your household have the following materials | | | | |  | Do you have own land? | 1. Yes 2. No |
|  | Electricity | | | 1. Yes 2. No |  | Number of member per bed room |  |
|  | Radio | | | 1. Yes 2. No | Does the household have the following? | | |
|  | Television | | | 1. Yes 2. No |  | Cows/bulls | 1. Yes 2. No |
|  | Telephone | | | 1. Yes 2. No |  | Horse/donkey/mules | 1. Yes 2. No |
|  | Computer | | | 1. Yes 2. No |  | Camels | 1. Yes 2. No |
|  | Refrigerator | | | 1. Yes 2. No |  | Goats | 1. Yes 2. No |
|  | Table | | | 1. Yes 2. No |  | Sheep | 1. Yes 2. No |
|  | Chair | | | 1. Yes 2. No |  | Chickens | 1. Yes 2. No |
|  | Bed with mattress | | | 1. Yes 2. No |  | Beehives | 1. Yes 2. No |
|  | Electric mitad | | | 1. Yes 2. No |  | Land area (m^2^) |  |
|  | Watch | | | 1. Yes 2. No | | | |
|  | Mobile phone | | | 1. Yes 2. No | | | |
|  | Bicycle | | | 1. Yes 2. No | | | |
|  | Motorcycle | | | 1. Yes 2. No | | | |
|  | Animal drawn cart | | | 1. Yes 2. No | | | |
|  | Cart or truck | | | 1. Yes 2. No | | | |
|  | Bajaj | | | 1. Yes 2. No | | | |
|  | Bank account | | | 1. Yes 2. No | | | |

**Part III: Household health and related characteristics**

1. Insurance status of your household? 1. Insured 2. None insured
2. Was any household member/s sought healthcare services the last 12 months? 1. Yes 2. No
3. If yes to Q1, how many members were sought cares? , list the table below.

| Code | Sex | Where care was sought (write code)   1. Home based 2. Governmental HF, 3. Private HF 4. Traditional | How many times and duration of all episodes?(write frequency and duration, f/d) | Reason to seek care? | Admission history?   1. Yes 2. No | Frequency/all admissions duration? |
| --- | --- | --- | --- | --- | --- | --- |
|  |  |  |  |  |  |  |
|  |  |  |  |  |  |  |
|  |  |  |  |  |  |  |
|  |  |  |  |  |  |  |
|  |  |  |  |  |  |  |
|  |  |  |  |  |  |  |
| … |  |  |  |  |  |  |

1. For the last 12 months, has any member of your household had history of referral for treatment? 1. Yes 2. No
2. If yes to Q4, fill the following table?

| Code | Refer to(write place) | Frequency | Reason for referral? | How many days you stay for one referral? | Total referral expense (treatment, transport, lodge and cafeteria) |
| --- | --- | --- | --- | --- | --- |
|  |  |  |  |  |  |
|  |  |  |  |  |  |
|  |  |  |  |  |  |
| … |  |  |  |  |  |

1. Is any household member having any chronic health conditions? 1. Yes 2. No
2. If yes to Q6, what type of chronic health conditions? More than one choice possible. 1. HTN 2. DM 3. Heart diseases 4. Renal diseases 5. Cancer 6. Others, specify…………………………………………………………………………………………

**Part IV: Total household healthcare expenditure measurement**

**I: Direct medical and Non-medical Healthcare costs (both outpatient and inpatient)**

| S/N | Questions | Possible answers |
| --- | --- | --- |
|  | How much money your household paid for all healthcare services received for the last 12 months? | 1. Registration card, ….…..……….birr 2. Medications ,… ………………birr 3. Diagnostic test (x-ray, CT, etc.) .birr. 4. laboratory,… …………………..birr 5. Bed, ……………………...birr |
|  | How much money your household paid for transport, cafeteria and lodging services for the last 12 months including caregivers cost? | 1. Transport, ………………………birr 2. Cafeteria ………………………..birr 3. Lodging, ……………………….birr |
| Total direct healthcare cost of the household | | Birr |

**II: Indirect healthcare cost (both outpatient and inpatient health care services)**

1. For how many days on average was each member seeking modern healthcare services absent from work/school because of the illness/healthcare services? Fill the lost days for each of members who have history of seeking care?

| Code | Total lost days | Average monthly income | Total indirect cost of each member sought care |
| --- | --- | --- | --- |
|  |  |  |  |
|  |  |  |  |
|  |  |  |  |
|  |  |  |  |
|  |  |  |  |
|  |  |  |  |
| … |  |  |  |
| Total indirect cost of household | | |  |

1. Has any caregiver accompany with while the household seeking healthcare services? 1. Yes 2. No
2. If yes to Q2, how many caregivers were accompanying with? ….caregivers. Fill the following table?

| Code | Total lost days for all accompany | Average monthly income | Total indirect cost of each caregiver |
| --- | --- | --- | --- |
|  |  |  |  |
|  |  |  |  |
|  |  |  |  |
|  |  |  |  |
|  |  |  |  |
|  |  |  |  |
| … |  |  |  |
| Total indirect cost of the household because of caregivers lost days | | |  |

**III: Direct and indirect Cost of traditional healthcare (for having history of seeking care from traditional healers)**

1. Did any household member seek healthcare service from traditional/religious center? 1. Yes 2. No
2. If yes to Q1, fill the table below

| Code | Total direct expenditure | | Total lost days | | Average monthly income | | Total cost |
| --- | --- | --- | --- | --- | --- | --- | --- |
|  | Care seeker | Caregiver | Care seeker | caregiver | Care seeker | Caregiver |  |
|  |  |  |  |  |  |  |  |
|  |  |  |  |  |  |  |  |
|  |  |  |  |  |  |  |  |
|  |  |  |  |  |  |  |  |
|  |  |  |  |  |  |  |  |
|  |  |  |  |  |  |  |  |
|  |  |  |  |  |  |  |  |
| Total household’s traditional/religious healthcare service | | | | | | |  |

**Part V: Household non-health expenditure and consumption**

| **I: Household average monthly food expenditure** | | |
| --- | --- | --- |
| S/N | List of foods and beverages needed per month | Expenditure (ETB) |
|  | Oil and fats |  |
|  | Cereals (maize grains, beans, rice, Misr) |  |
|  | Livestock/ Poultry products( e.g. Milk and eggs) |  |
|  | Meat including Doro wot |  |
|  | Sugar and coffee (tea, coffee) |  |
|  | Bread(wheat flour) and injera (teff) |  |
|  | Spices and salt (berbere, kimemakimem, ) |  |
|  | Vegetables and fruits(potato, tomato, banana) |  |
|  | Alcoholic drinks, Soft drinks and juice |  |
|  | Meals (hotel , restaurant, cafeteria) |  |
| **Total average monthly food expenditure** | |  |
| **II: Monthly household expenditure (nonfood)** | | |
| How much did your household spend in last one month on the following? | | |
|  | Cosmetics |  |
|  | Soap and detergent |  |
|  | Hair dressing/barber |  |
|  | Rent |  |
|  | Electricity |  |
|  | Water |  |
|  | Kerosene/paraffin |  |
|  | Telephone bills/Airtime |  |
|  | Transport |  |
|  | Charcoal |  |
|  | Fire wood |  |
|  | Cooking gas |  |
|  | Salaries including salaries/wages for domestic workers |  |
|  | Sanitary materials(dipper, modes, towels) |  |
|  | Others (Specify) |  |
|  | Total amount |  |
| **III: Annual household expenditure (nonfood)** | | |
| How much did your household spend in the last one year on the following? | | |
|  | Education (registration, uniforms, books, tuition, exam fees) |  |
|  | Maintenance and repairs including car and buildings etc. |  |
|  | Clothing and footwear |  |
|  | Social Affairs (Wedding/Funerals…) |  |
|  | Capital expenditures including cars, plots etc. |  |
|  | Others (specify) |  |
|  | Total amount |  |
| **Total nonfood expenditure** | |  |

**Part VI: Coping mechanisms of financial hardship**

| No | Question | Answer | Skip |
| --- | --- | --- | --- |
|  | What was the main source of fund to cover the household health costs | - - - 1. Own       2. Borrowing       3. Selling household assets       4. My family/relatives support       5. Others_______ | If 3 skip to 2. |
|  | If you borrowed, how much you borrow? | ________birr |  |
|  | From whom did you borrow? | 1. Family 2. Cooperative  3 Neighbors/friends 4. Private  4. Others, specify……………….. |  |
|  | If you sold your household asset, what kind of asset you have sold? | 1. Household item 2. Jewelry  3. Vehicle 4. House  5. Land 6. Others specify……………… |  |

Thank You so much!!!
