# Supplementary material for: Leaving no one behind in health: Financial hardship to access health care in Ethiopia
Source: PLoS One. 2023 Mar 13;18(3):e0282561. doi: 10.1371/journal.pone.0282561 (PMC10010508; doi:10.1371/journal.pone.0282561)
Supplement: S2 Text — (DOCX) [file pone.0282561.s002.docx]

## S2 Text: Survey questionnaire (Amharic version)

የቤቱ ኮድ____________ቃለ መጠይቁ የተደረገበት ቀን____________

የጠያቂው ስም ________________ ፊርማ____________

የተመራማሪው ስም______________ ፊርማ____________

**ክፍል 1፡ የስነ ሕዝብ አወቃቀር እና የማኅበረሰቡ ኢኮኖሚያዊ ባህሪያት**

1. የቤተሰብ አስተዳዳሪው ፆታ? 1. ወንድ 2. ሴት

2. የቤተሰብ አስተዳዳሪው ዕድሜ? በአመት ____________

3. የቤተሰብ አስተዳዳሪው የሚከተለው ሃይማኖት? 1. ኦርቶዶክስ 2. ሙስሊም 3. ሌሎች፣ ይግለፁ…………

4. የቤተሰብ አስተዳዳሪው ብሄር? 1. አማራ 2. ኦሮሞ 3. ትግሬ 4. ሌሎች፣ ይግለፁ…………

5. የቤተሰብ አስተዳዳሪው የትዳር ሁኔታ? 1. ያላገባ 2. ያገባ 3. ተለያይቶ እሚኖር 4. የተፋታ/ች 5. የሞተበት/ባት

6. የቤተሰብ አስተዳዳሪው የትምህርት ደረጃ? 1. መፃፍ እና ማንበብ እማይችል 2. ማንበብ እና መፃፍ ግን መደበኛ ትምህርት ያልተማረ 3. የመጀመሪያ ደረጃ (1-8) 4. ሁለተኛ ደረጃ (9-12) 5. ኮሌጅ / ዩኒቨርሲቲ እና ከዚያ በላይ

7. የቤቱ አስተዳዳሪ የሥራ ዘርፍ? 1. ሥራ አጥ 2. በግል ሥራ የሚተዳደር 3. በመንግሥት የተቀጠረ 4. በግል ድርጅቶች የተቀጠረ 5. ሌላ ካለ ይግለፁ……

8. የቤቱ አባውራ አማካይ ወርሃዊ ገቢ ስንት ነው? ………………ብር

9. የቤተሰብ አባላት ብዛት ስንት ነው (አባውራውን ጨምሮ)? ሰንጠረዡን ይሙሉ (አባውራ አይጨምርም)።

| ኮድ | ዝምድና(ከአባውራ ጋር) | እድሜ | ፆታ | ሀይማኖት | የትም/ት ደረጃ | የስራ ዘርፍ | ወርሃዊ አማካኝ ገቢ |
| --- | --- | --- | --- | --- | --- | --- | --- |
| M1 |  |  |  |  |  |  |  |
| M2 |  |  |  |  |  |  |  |
| M3 |  |  |  |  |  |  |  |
| M4 |  |  |  |  |  |  |  |
| M5 |  |  |  |  |  |  |  |
| M6 |  |  |  |  |  |  |  |
| M7 |  |  |  |  |  |  |  |
| M8 |  |  |  |  |  |  |  |
| M9 |  |  |  |  |  |  |  |
| M10 |  |  |  |  |  |  |  |
| ድምር |  |  |  |  |  |  |  |

**ክፍል 2፡ የቤት ሀብት ሁኔታ መለኪያ መጠይቆች**

| ተ/ቁ | | ጥያቄ | መልስ | | ተ/ቁ | ጥያቄ | መልስ |
| --- | --- | --- | --- | --- | --- | --- | --- |
|  | | የቤቱ ባለቤትንት | 1. የግል 2. የኪራይ | |  | የቤቱ ወለል የተሰራበት ቁሳቁስ? | 1. የተፈጥሮ (አፈር  2. ኮንክሪት  3. ሴራሚክስ  4. ሌሎች ይገልጻሉ….. |
|  | | የቤቱ ዋና የመጠጥ ውሀ ምንጭ? | 1. የቧንቧ ውሃ በቤት ውስጥ  2. የተጠበቀ የጉድጓድ ውሃ  3.ያልተጠበቀ የጉድጓድ ውሃ  4. ጥብቅ ምንጭ  5. ሌላ ካለ………….. | |  |  |  |
|  | | የሽንት ቤቱ አይነት? | 1. የለም  2. ባህላዊ መጸዳጃ ቤት  3. ማስተንፈሻ ያለው  4. ውሃ በማፍሰስ እሚሰራ  5. ሌላ ካለ……………… | |  | የቤቱ ጣሪያ የተሰራበት ቁሳቁስ?? | - - - 1. ከሳር       2. ከቆርቆሮ       3. ሌላ ካለ______ |
|  | | ዋና የምግብ ማብሰያ ዓይነት? | 1. እንጨት 2. ከሰል  3. ባዮጋዝ 4. ኬሮሴን 5. ኤሌክትሪክ | |  | የቤቱ ግድግዳ የተሰራበት ቁሳቁስ? | - - - 1. ከእነጨትና ከጭቃ       2. ከሲሚንቶ       3. ከሴራሚክ       4. ሌላ ካለ______ |
| በቤታችሁ ውስጥ የሚከተሉት ነገሮች አሉ? | | | | |  | የራስህ መሬት አለዎት? | 1. አለ 2. የለም |
|  | ኤሌክትሪክ | | | 1. አለ 2. የለም |  | በአንድ መኝታ ክፍል እሚተኙ ሰዎች ብዛት |  |
|  | ሬዲዮ | | | 1. አለ 2.የለም | በቤታችሁ ውስጥ የሚከተሉት ነገሮች አሉ? | | |
|  | ቴሌቪዥን | | | 1. አለ 2. የለም |  | ላም/በሬ | 1. አለ 2. የለም |
|  | የቤት ስልክ | | | 1. አለ 2. የለም |  | ፈረስ/በቅሎ/አህያ | 1. አለ 2. የለም |
|  | ኮምፒውተር | | | 1. አለ 2. ለም |  | ግመል | 1. አለ 2. የለም |
|  | ፍሪጅ | | | 1. አለ 2. የለም |  | ፍየል | 1. አለ 2. የለም |
|  | ጠረጴዛ | | | 1. አለ 2. የለም |  | በግ | 1. አለ 2. የለም |
|  | ወንበር | | | 1. አለ 2. የለም |  | ዶሮ | 1. አለ 2. የለም |
|  | አልጋና ፍራሽ | | | 1. አለ 2. የለም |  | የንብ ቀፎ | 1. አለ 2. የለም |
|  | ኤሌክትሪክ ምጣድ | | | 1. አለ 2. የለም |  | የመሬት ስፋት(በሜ^2^ ) |  |
|  | የግድግዳ ሰዓት | | | 1. አለ 2. የለም | | | |
|  | ሞባይል ስልክ | | | 1. አለ 2. የለም | | | |
|  | ብስክሌት | | | 1. አለ 2. የለም | | | |
|  | ሞተር ሳይክል | | | 1. አለ 2. የለም | | | |
|  | የእንሰሳት ጋሪ | | | 1. አለ 2. የለም | | | |
|  | የጭነት መኪና | | | 1. አለ 2. የለም | | | |
|  | ባጃጅ | | | 1. አለ 2. የለም | | | |
|  | የባንክ ሒሳብ ቁጥር | | | 1. አለ 2. የለም | | | |

**ክፍል 3: የቤተሰብ ጤና እና ተዛማጅ ሁኔታዎች**

1. ባለፉት 12 ወራት በቤተዎ ውስጥ ማንኛውንም የጤና አገልግሎት የተሰጠው ነበር? 1. አዎ 2. አይደለም

2. ለጥያቄ ቁጥር 1 አዎ ከሆነ፣ ስንት የቤተስብ አባላትአግልግሎቱን ተሰጠዋል? ሰንጠረዡን ይሙሉ

| ኮድ | አገልግሎት ያገኙበት የት ነው (ኮዱን ይፃፉ)   1. ከቤት 2. ከመንግስት ጠቋም 3. ከግል ጤና ጠቋም 4. ከባህል/ሀይማኖታዊ | ለስንት ጊዜና በሁሉም ድግግሞሽ ስንት ቀን ከስራ ከለከለዎት(ድ/የቀን ብዛት) | አገልግሎት የፈለጉበት በምን ምክንያት ነው? | ተኝተው ታክመዋል?   1. አዎ 2. አለታከምኩም   (ኮዱን ይፃፉ | ተኝተው የታከሙበት ድግግሞሽ/በሁሉም ድግግሞሽ የቆዩበት ቀን ብዛት  (ድግግሞሽ/የቀን ብዛት) |
| --- | --- | --- | --- | --- | --- |
|  |  |  |  |  |  |
|  |  |  |  |  |  |
|  |  |  |  |  |  |
|  |  |  |  |  |  |
|  |  |  |  |  |  |
|  |  |  |  |  |  |
| … |  |  |  |  |  |

3. ከታመሙት የቤተሰብ አባላት ውስጥ ላለፉት 12 ወራት ሪፈር የተላከ አባል አለ? 1. አዎ 2. የለም

4. ለጥያቄ ቁጥር 3 አዎ ከሆነ፣ የሚከተለውን ሠንጠረዥ ይሙሉ?

| ከድ | ሪፈር የተባሉበት ቦታ | ድግግሞሽ | ሪፈር የተባሉበት ምክንያት? | በሪፈር ምክናያት ስንት ቀን ከስራ ቀሩ? | አጠቃለይ ል ሪፈር ያወጡት የብር መጠን (ህክምና, ምጓጓዣ, አለጋና ካፍቴሪያ) |
| --- | --- | --- | --- | --- | --- |
|  |  |  |  |  |  |
|  |  |  |  |  |  |
|  |  |  |  |  |  |
|  |  |  |  |  |  |

5. ለረጅም ጊዜ የቆየ የጤና ችግር ያለበት የቤተሰብ አባል አለ? 1. አዎ 2. አይደለም

6. ለጥያቄ ቁጥር 5 አዎ ከሆነ ምን አይነት በሽታ/ሁኔታ፤ ከአንድ በላይ ምርጫ ይቻላል. 1. የደም ግፊት 2. የስኳር በሽታ 3. የልብ ሕመም 4. የኩላሊት ህመም 5. ካንሰር 6. ሌሎች ካሉ ይግለጹ……………………

**ክፍል 4፡ ጠቅላላ የቤተሰብ ጤና አጠባበቅ ወጪ መለኪያ**

**ሀ፡ ቀጥተኛ የህክምና እና የህክምና ያልሆኑ የጤና አጠባበቅ ወጪዎች (የተመላላሽ እና የታካሚ)**

| S/N | ጥያቄዎች | መልሶች |
| --- | --- | --- |
|  | የእርስዎ ቤተሰብ ላለፉት 12 ወራት ላገኙት ለሁሉም አይነት የጤና ጥበቃና አገልግሎቶች ምን ያህል ገንዘብ ከፍለዋል? | 1. የመመዝገቢያ ካርድ፣ ………………ብር 2. መድሃኒቶች ……………………….. ብር 3. ለምስል ምርመራ (ኤክስሬይ.) ………ብር 4. ለላብራቶሪ……………………….. ብር 5. አልጋ፣ …………………………. …ብር |
|  | ቤተሰብዎ ለአለፉት 12 ወራት ለትራንስፖርት፣ ለካፍቴሪያ እና ለአልጋ አገልግሎት የአስታማሚን ወጪዎች ጨምሮ ምን ያህል ገንዘብ ከፍለዋል? | 1. . ትራንስፖርት፣ ……………………ብር 2. ካፊቴሪያ፣ ………………………… ብር 3. ማረፊያ፣ …………………………ብር |
| ድምር | | ብር |

**ለ፡ ኢ-ቀጥተኛ የጤና አገልግሎት ወጪ**

- - - 1. ባለፈው አንድ አመት ዘመናዊ የጤና አገልግሎት ያገኘው/ችው የቤተሰብ አባል በህመም/በጤና አገልገሎት ምክንያት በአማካይ ለስንት ቀናት ከስራ/ትምህርት ቀርቷል?

| Code | የቀናት ብዛት | አማካይ ወራዊ ገቢ ((በብር) | ጠቅላላ ኢ-ቀጥተኛ የጤና ወጭ |
| --- | --- | --- | --- |
|  |  |  |  |
|  |  |  |  |
|  |  |  |  |
|  |  |  |  |
|  |  |  |  |
|  |  |  |  |
| … |  |  |  |
| ጠቅላላ የቤተሰቡ ኢ-ቀጥተኛ የጤና ወጭ | | |  |

- - - 1. የጤና አገልግሎት ለማግኘት ጤና ተቋም በሚሄዱበት ወቅት አብሮዎት የሄደ ሰው ነበር? 1. አዎ 2. የለም
      2. ለጥያቄ ቁጥር 2 አዎ ከሆነ፣ ስንት ሰዎች ናቸው አብረው የሄዱት?.....የሚከተለውን ሰንጠረዥ ይሙሉ፡፡

| Code | የቀናት ብዛት | አማካይ ወራዊ ገቢ (በብር) | ጠቅላላ የተንከባካቢ ኢ-ቀጥተኛ የጤና ወጭ |
| --- | --- | --- | --- |
|  |  |  |  |
|  |  |  |  |
|  |  |  |  |
|  |  |  |  |
|  |  |  |  |
|  |  |  |  |
| … |  |  |  |
| ጠቅላላ የሁሉም ተንከባካቢ ኢ-ቀጥተኛ የጤና ወጭ | | |  |

**ሐ፡ ለባህላዊ/ሀይማኖት ነክ የጤና አገልግሎት የሚወጣ ቀጥተኛና ኢ-ቀጥተኛ ወጭ(የባህላው ህክምና አገልግሎት ላላቸው ብቻ**

1. ባለፈው አንድ አመት ውስጥ ከቤተሰብዎ ውስጥ የባህላዊ/ሀይማታዊ የጤና አገልግሎት ያገኘ ነገር? 1. አዎ 2. የለም
2. መልሱ አዎ ከሆነ የሚከተለውን ሰንጠረዥ ይሙሉ፡፡

| ኮድ | ጠቅላላ ቀጥተኛ ወጭ | | ጠቅላላ ኢ-ቀጥተኛ ወጭ | | አማካይ ወራዊ ገቢ (በብር) | | ጠቅላላ ወጭ |
| --- | --- | --- | --- | --- | --- | --- | --- |
|  | ታካሚው | እንክብካቤ ሰጭው | ታካሚው | እንክብካቤ ሰጭው | ታካሚው | እንክብካቤ ሰጭው |  |
|  |  |  |  |  |  |  |  |
|  |  |  |  |  |  |  |  |
|  |  |  |  |  |  |  |  |
|  |  |  |  |  |  |  |  |
|  |  |  |  |  |  |  |  |
|  |  |  |  |  |  |  |  |
|  |  |  |  |  |  |  |  |
| ጠቅላላ የባህላው ጤና አገልግሎት ወጭ(በብር) | | | | | | |  |

**ክፍል 4: ከጤና ውጭ የሆነ የቤት ወጭ**

| **ሀ: የቤት ወራዊ አማካይ የምግብ ወጭ** | | | |
| --- | --- | --- | --- |
| ተ/ቁ | የምግበ ዝርዝር | ወጭ (በብር) | |
|  | ለዘይትና ለቅባት እህሎች |  | |
|  | ለጥራጥሬ |  | |
|  | ለእንሳት ተዋፅኦ(እንቁላል፣ ወተት) |  | |
|  | ስጋ ዶሮ ወጥን ጨምሮ |  | |
|  | ለስኳርና ለቡና |  | |
|  | ለዳቦና ለጤፍ |  | |
|  | ለበርበሬ፣ለጨውና ለቅመማቅመም |  | |
|  | ለአትክልትና ፍራፍሬ |  | |
|  | ለአልኮልና ለለስላሳ መጠጦች |  | |
|  | ውጭ ላይ ለመመገብ(ካፌ፣ ሆቴልና ሬስቶራንት) |  | |
| **ጠቅላላ ወራዊ የምግብ ወጭ** | |  | |
| **ለ: ወራዊ ከምግብ ውጭ የሆኑ ወጭዎች** | | | |
| ባለፈው አንድ ወር ውስጥ ለሚከተሉት ፍጆታዎች ቤተዎ ስንት ብር ያወጣል | | | |
|  | ለውበት መጠበቂያ |  | |
|  | ለሳሙናና ዲተርጀንት |  | |
|  | ፀጉርን ለመሰራት(ለመቆረጥ) |  | |
|  | ለቤት ኪራይ |  | |
|  | ለመብራት |  | |
|  | ለውሃ |  | |
|  | ለጋዝ(ኬሮሰን) |  | |
|  | ለስልክ |  | |
|  | ለመጓጓዣ |  | |
|  | ለማገዶ እንጨትና ለከሰል |  | |
|  | ለማብሰያ ጋዝ |  | |
|  | ለሰራተኛ ደመወዝ |  | |
|  | ለፅዳትና ንፅህና መጠበቂያ(ፎጣ፣ ሞዲየስ) |  | |
|  | ሌላ ወጭ ካለ ይገለፁ |  | |
|  | ጠቅላለ ድምር |  | |
| **ሐ: አመታዊ የቤት ወጭ** | | | |
| ባለፈው አንድ አመት ውስጥ ለሚከተሉት ፍጆታዎች ቤተዎ ስንት ብር አወጣ? | | | |
|  | ለትምህርት (ለመመዝገቢያ, የደንብ ልብስ, መፅሐፍት, የትምህርት ክፍያ) | |  |
|  | ለቤት/ለመኪና ጥገና | |  |
|  | ለልብስና ለጫማ | |  |
|  | ለማህበራዊ ጉዳዮች(ለቀብር፣ ለሰርግ፣) | |  |
|  | ለካፒታል ወጭ | |  |
|  | ሌሎች ካሉ ይግለፁ | |  |
|  | ጠቅላላ ድምር | |  |
| **አጠቃላይ ከምግብ ውጭ የሆኑ አመታዊ ወጭዎች** | | |  |

**ክፍል 4: የጤናን ወጭ መቋቋሚያ መንገድ**

| ተ/ቁ | ጥያቄ | መልስ | ዝለል |
| --- | --- | --- | --- |
|  | የጤና አገልግሎት ወጭዎን ከየት ነዉ የሚያገኙት? | - - - 1. ከራሰ ገንዘብ (ከደሞዝ, ከቁጠባ)       2. ገንዘብ ተበድሬ/ከብድር       3. የቤት ንብረቴን ሸጬ ነው       4. ከማህበራዊ እርዳታ       5. ሌላ ካለ ይግለፁ………………….. | መልሰዎ 3 ወደ ተራ ቁጥር 4 ይዝለሉ. |
|  | ተበድረው ከሆነ ስንት ብር ተበደሩ? | ___________________ብር |  |
|  | ተበድረው ከሆነ ከማነው የተበደሩት? | ከቤተሰብ  ከግል ድርጅት  ከጎረቤት/ከጓደኛ  በትበብር የሚደረግ  ሌላ ካለ ይግለፁ………………………… |  |
|  | የበት ንብረተዎን ሽጠው ከሆነ ምንድን ነው የሸጡት? | 1. የቤት ዕቃ 2. ጌጣጌጥ  3. ተሸከርካሪ /መኪ 4. ቤት  5. መሬት  6. ሌላ ካለ ይግለፁ………………………….. |  |

በጣም አመሰግናለሁ!
