# Supplementary material for: Leaving no one behind in health: Financial hardship to access health care in Ethiopia
Source: PLoS One. 2023 Mar 13;18(3):e0282561. doi: 10.1371/journal.pone.0282561 (PMC10010508; doi:10.1371/journal.pone.0282561)
Supplement: Attachment — Submitted filename: comments on leave no one behind.docx [file pone.0282561.s003.docx]

Comments
The paper is about “Leaving no one behind in health: financial hardship to access health care in Ethiopia” which is very important and interesting research area for Ethiopia and beyond, as it is clearly shown in the introduction part of the manuscript that financial hardship (risk) protection is the international and national agenda, such as SDGs and Health Sector Transformational Plan two(HSTPII), which is a five year(2021-2025) plan (part of the long Ethiopia’s health sector’s fifteen years strategic plan (2015-2035)).As per the findings of this study, due to financial hardship, the poor people(found in low income countries including Ethiopia) either forgone and/or delayed for accessing health care they need or they are obligated to access the needy health care on the expense of other basic needs like food which is against the zero hunger goal of the agenda 2030. In the era of Covid-19 pandemic and other disasters (e.g. conflict and climate change), the poor left behind in health (care).The findings of this study indicated that financial hardship becomes a significant barrier to achieve the notion of leave no behind. For example, impoverished health care makes, about 10.4%, households became poor and catastrophic health expenditure was found in 37.1% of the households.

Hence, it is very relevant and suitable to publish this evidence that showed the extent of financial hardship (and the associated factors) on households in Ethiopia in general and in Debre Tabor town (one of conflict affected areas of Ethiopia) in particular. This evidence will inform policy options in the country in order to attain the universal health coverage (leave no one behind in health).Despite, there are few comments that need to be either clear or addressed before this paper could be published.
1. The threshold that the authors used for catastrophic health expenditure is 10%. However, they put the results in three thresholds (10%, 25% and 40% non-food). Why this is so?
2. In the introduction and discussion sections of the manuscript, there are long sentences that can be reconstructed to simple, short and concise sentences as per the plos one journal recommendation.

3. The authors should convert the Ethiopia Birr to US Dollar in bracket to make it clear for international community.

4. in the methods section your dependent variable says Financial hardship of healthcare(CHE ) : here the abbreviation CHE is not represent financial hard ships of health care so use either of the two / catastrophic health expenditure or financial hard ship

5. In the measurement of catastrophic health expenditure as you clearly present the threshold ranges from 10%- 40 %. So why you select the lower value(10%)? Because of this decision you may under estimate the level of catastrophic helth expenditure in the study area?
