# Supplementary material for: Leaving no one behind in health: Financial hardship to access health care in Ethiopia
Source: PLoS One. 2023 Mar 13;18(3):e0282561. doi: 10.1371/journal.pone.0282561 (PMC10010508; doi:10.1371/journal.pone.0282561)
Supplement: Attachment — Submitted filename: Response to Reviewers.docx [file pone.0282561.s004.docx]

**Response to editor:**

We really thank you, editor, for your insightful comments that are vital to improve the manuscript. We accept all of your comments and we corrected accordingly.

Here, below, are the responses for the comments:

1. “It is multiple logistic regression, not multivariate logistic regression. So, please change it throughout the manuscript”.

- Yes, you are quite right, we changed to multiple logistic regression throughout the manuscript.

2. “Before modelling factors using multiple logistic regression you need to develop a conceptual framework of your own or use an established framework from the literature. This conceptual model will serve as hypotheses that will guide your modelling process. For example, you may find some factors not statistically significant but they could be theoretically important for the Ethiopian contexts and you need to retain those factors in your final parsimonious model although they are not statistically significant”.

- We have developed the conceptual framework, but not reported in the 1^st^ version of the manuscript. Now, we have reported in the manuscript.

3. “Your interpretation of OR is wrong. You interpreted OR like RR. OR 2.5 means 2.5 times higher odds, not 2.5 times more likely!”

- Yes, you are correct, in the current version, we have corrected accordingly.

**Response to reviewers**

Thank you, reviewers, for your constructive comments that are vital to improve the manuscript. We accept all of your comments and we corrected accordingly.

Here, below, are the responses for the comments and questions raised:

1. “The threshold that the authors used for catastrophic health expenditure is 10%. However, they put the results in three thresholds (10%, 25% and 40% non-food). Why this is so?”

- Most authors, including in World health organization and World Bank joint report on UHC, used 10% and 25% thresholds. The 40% non-food also used by authors as well, as stated in the methods part of the manuscript. Therefore, we used the 10% threshold for statistical modeling which is the WHO threshold. However, we stated (in the manuscript) that the incidence CHE with the three thresholds (10%, 25% and 40% non-food) to allow the readers and/or reviewers to compare the incidence with these three thresholds. This will give more information than using only 10% threshold.

2. “In the introduction and discussion sections of the manuscript, there are long sentences that can be reconstructed to simple, short and concise sentences as per the plos one journal recommendation”.

- We have reconstructed accordingly.

3. “The authors should convert the Ethiopia Birr to US Dollar in bracket to make it clear for international community”

- We have converted in to $ in bracket as per the comment.

4. “In the methods section your dependent variable says Financial hardship of healthcare (CHE): here the abbreviation CHE is not represent financial hardships of health care so use either of the two / catastrophic health expenditure or financial hard ship”.

- We corrected as per the comment

5. “In the measurement of catastrophic health expenditure as you clearly present the threshold ranges from 10%- 40 %. So why you select the lower value (10%)? Because of this decision you may under estimate the level of catastrophic health expenditure in the study area?”

- This is because 10% is the usual threshold that used in global reports, including WHO and WB. Hence, the audience can compare the findings with that of such reports (e.g WHO reports on UHC). However, we have reported the incidence/headcount at 25% and 40% non-food as well to give additional information at these thresholds.
